# Impact of obesity on clinical outcomes of v-NOTES hysterectomy: a retrospective study

**DOI:** 10.3389/fmed.2025.1619188

**Published:** 2025-08-29

**Authors:** Liu Yang, Wang Qilin, Lian Haiying, Pan Feng, Li Tao, Li Junqiang

**Affiliations:** ^1^Affiliated Hospital of Southwest Jiaotong University, The Third People’s Hospital of Chengdu, Chengdu, China; ^2^West China Longquan Hospital Sichuan University, The First People’s Hospital of Longquanyi Chengdu District, Chengdu, China; ^3^Affiliated Hospital of Southwest Medical University, Luzhou, China

**Keywords:** obesity, vaginal natural orifice transluminal endoscopic surgery, total hysterectomy, postoperative recovery, retrospective study

## Abstract

**Background:**

Obesity is a global health challenge that complicates gynecological surgery. Vaginal natural orifice transluminal endoscopic surgery (v-NOTES) offers a minimally invasive approach to total hysterectomy (TH), but its safety and efficacy in obese patients remain underexplored.

**Objective:**

This study aimed to assess the impact of obesity on the perioperative and clinical outcomes of v-NOTES hysterectomy, accounting for potential confounders.

**Methods:**

This retrospective cohort study analyzed 211 patients who underwent v-NOTES TH between January 2021 and September 2024. Patients were categorized into two groups based on BMI: the control group (BMI < 28 kg/m^2^, *n* = 112) and the obesity group (BMI ≥ 28 kg/m^2^, *n* = 99). Intraoperative indicators and postoperative outcomes during hospitalization, including operative time, intraoperative blood loss, gastrointestinal recovery, hospital stay, and postoperative complications, were compared. A multivariable regression analysis was used to adjust for confounders. All patients were followed up during hospitalization and at 2 and 6 weeks postoperatively.

**Results:**

Obese patients had significantly longer operative times (*β* = 39.2, *p* < 0.001), delayed gastrointestinal recovery (time to first flatus: *β* = 5.8, *p* = 0.018), and prolonged hospital stays (*β* = 1.3, *p* = 0.002). No significant differences were found in intraoperative blood loss, conversion rates, blood transfusion, postoperative complication rates, or total hospitalization costs (limited to the inpatient period; all *p* > 0.05).

**Conclusion:**

v-NOTES hysterectomy is a safe and effective option for obese patients, with comparable complication rates to non-obese patients. However, obesity independently contributes to longer operative times and delayed recovery. Targeted perioperative strategies, particularly for improving gastrointestinal recovery, could enhance outcomes in this population.

## 1 Introduction

Obesity is a global epidemic and a significant risk factor for various surgical complications, including prolonged operative time, increased blood loss, and delayed recovery (1, 2). In gynecological surgery, these challenges are particularly pronounced due to the anatomical and technical difficulties associated with obesity (3).

Vaginal natural orifice transluminal endoscopic surgery (v-NOTES) has emerged as a minimally invasive technique combining the advantages of vaginal and laparoscopic approaches. By leveraging a natural orifice, v-NOTES avoids abdominal incisions, potentially reducing postoperative pain, scarring, and recovery time (4, 5). However, evidence regarding the safety and efficacy of v-NOTES in obese patients is limited, as most published studies have focused on conventional laparoscopic or robotic approaches (6, 7).

Notably, the versatility and broader applicability of the vaginal route in minimally invasive surgery have also been emphasized in general surgical contexts (8). This finding supports its potential value in high-risk populations, such as obese patients undergoing gynecologic procedures.

This study aims to evaluate the perioperative outcomes of v-NOTES hysterectomy in obese versus non-obese patients, addressing a critical gap in the literature. The findings will contribute to clinical decision-making and optimization of surgical strategies for high-risk populations.

## 2 Methods

### 2.1 Study design and population

This retrospective observational study adhered to the Strengthening the Reporting of Observational Studies in Epidemiology (STROBE) guidelines for observational research. Clinical data were collected from patients who underwent total hysterectomy (TH) via v-NOTES between January 2021 and September 2024 at three tertiary referral hospitals in Sichuan Province, China: the Affiliated Hospital of Southwest Jiaotong University, the Affiliated Hospital of Southwest Medical University, and Longquan Hospital of West China Hospital, Sichuan University. All procedures were performed by experienced gynecologic surgeons who had completed over 50 v-NOTES hysterectomies prior to the initiation of this study.

### 2.2 Inclusion and exclusion criteria

Patients were eligible for inclusion if they underwent TH for benign gynecological conditions using the v-NOTES surgical approach and had no evidence of pelvic organ prolapse. Patients were excluded if they had incomplete clinical data, severe comorbidities (e.g., cardiac or respiratory failure), or if postoperative pathology revealed malignancies requiring additional treatment.

### 2.3 Patient selection and grouping

A total of 250 patients were initially screened for eligibility. Based on the inclusion and exclusion criteria, 39 patients were excluded: 22 due to incomplete clinical data, 7 due to malignancy diagnosed postoperatively, and 10 due to significant comorbidities that could confound perioperative outcomes. These comorbidities included patients with poorly controlled diabetes requiring prolonged preoperative insulin adjustment (*n* = 3), severe hypertension requiring inpatient stabilization (*n* = 1), recent heart valve replacement on anticoagulation therapy with perioperative bridging (*n* = 2), and concurrent non-gynecologic surgical procedures such as lipoma excision (*n* = 4).

Ultimately, 211 patients were included in the final analysis. They were stratified into two groups based on their body mass index (BMI): the control group (BMI < 28 kg/m^2^, *n* = 112) and the obesity group (BMI ≥ 28 kg/m^2^, *n* = 99). The BMI range was 18.5–27.9 28 kg/m^2^ in the control group and 28.0–39.5 kg/m^2^ in the obesity group.

**Figure 1:**
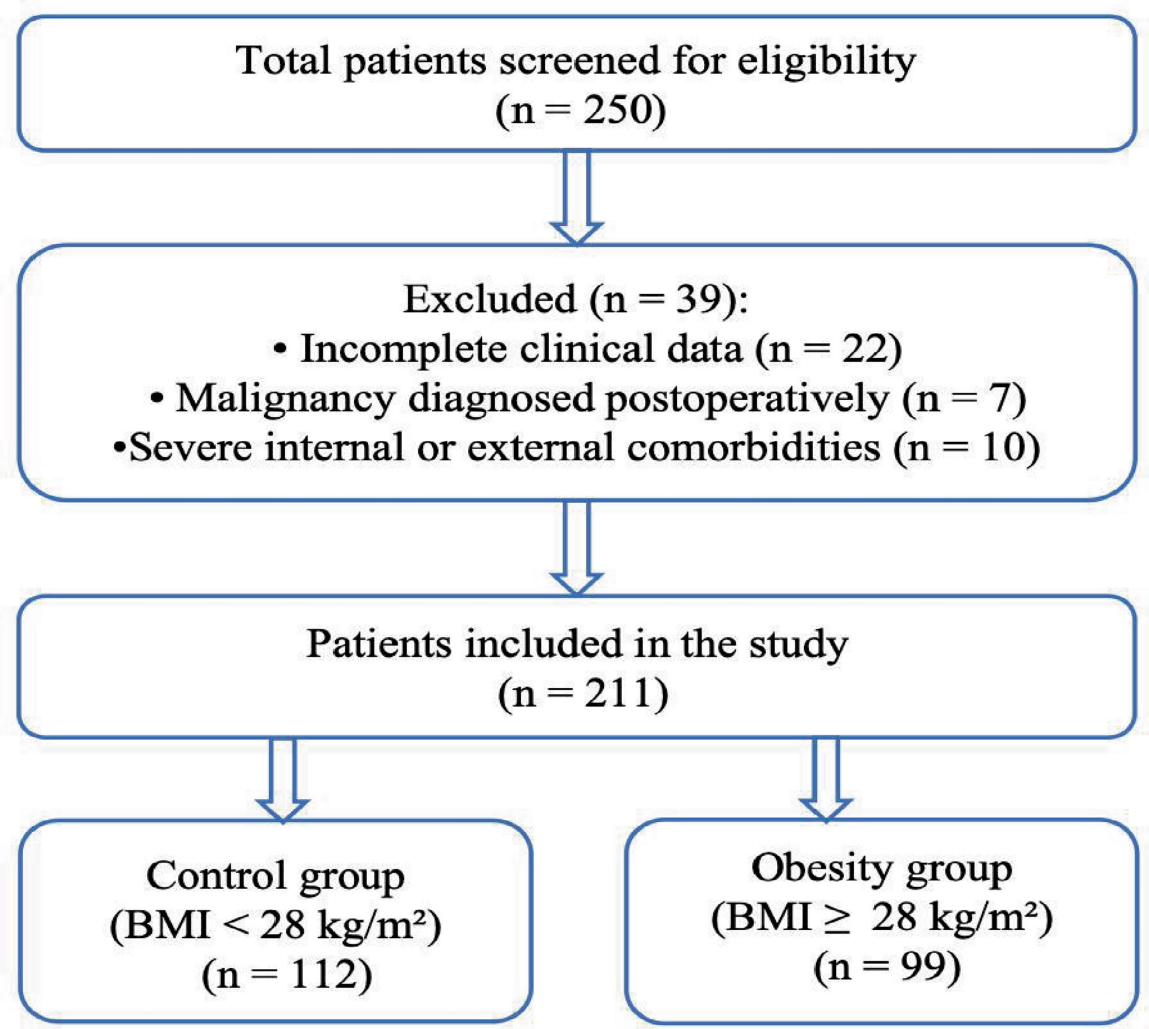
Patients’ selection flowchart.

The flow of patient selection and grouping is summarized in Figure 1.

## 3 Study variables

### 3.1 Baseline characteristics

Baseline characteristics included patient age, history of vaginal delivery, history of abdominal surgeries, uterine weight, and surgical indications. Uterine weight was estimated using the formula: uterine weight (g) = Uterine density (1.04 g/cm^3^) × Uterine volume. Uterine volume was calculated as follows:


$$\begin{array}{l} 4/3\times \pi \times (\text{length}/2)\times (\text{anteroposterior diameter}/2)\times \\ \left(\text{transverse diameter}/2\right) \end{array}$$

### 3.2 Intraoperative variables

Intraoperative variables analyzed in this study included operative time (minutes), estimated blood loss (mL), the number of cases requiring conversion to other surgical approaches, and the requirement for intraoperative blood transfusion.

### 3.3 Perioperative variables

Perioperative outcomes included time to first flatus (hours), incidence of urinary retention, number of patients who developed postoperative fever, hemoglobin (Hb) drop (calculated as the difference between preoperative Hb and Hb on postoperative day 2), length of hospital stay (days), and total hospitalization costs (measured in CNY and limited to the inpatient period).

Additionally, we reviewed medical records and conducted follow-up at 2 and 6 weeks postoperatively to assess early complications, including infections, hematoma, and intraoperative injuries; no major complications were reported after discharge.

## 4 Statistical analysis

### 4.1 Descriptive statistics

Categorical variables were expressed as percentages (%) and compared using the chi-square test. Continuous variables were presented as mean ± standard deviation (X ± SD) and compared between groups using independent samples t-tests. For comparisons among three groups (e.g., different obesity levels), a one-way analysis of variance (ANOVA) was applied if the data were normally distributed and met the assumption of homogeneity of variance. If these assumptions were not met, the non-parametric Kruskal–Wallis H test was used instead.

### 4.2 Multivariable adjustment

To account for potential confounding factors such as age, uterine weight, and surgical indications, a multivariable linear regression analysis was performed for continuous outcomes, and a logistic regression analysis was applied for binary outcomes. The results of linear regression were reported as *β* coefficients with 95% confidence intervals (CIs), while logistic regression results were expressed as adjusted odds ratios (aORs) with 95% CI.

### 4.3 Sample size considerations

No formal sample size calculation was conducted due to the retrospective nature of the study. The sample size was determined by the number of eligible patients within the study period (*n* = 211). While sufficient for detecting major differences between the control and obesity groups, the statistical power needed to detect smaller differences may be limited.

### 4.4 Statistical software

All statistical analyses were performed using SPSS 23.0 software. A *p*-value of < 0.05 was considered statistically significant.

## 5 Bias control

To minimize selection bias, all eligible patients meeting the inclusion criteria within the study period were included. Standardized data collection protocols were used to reduce information bias. Additionally, multivariable regression analysis was used to adjust for confounding factors, and sensitivity analyses were conducted to assess the robustness of the findings.

## 6 Results

### 6.1 Baseline characteristics

A total of 211 patients were included in the study, with 112 patients in the control group and 99 patients in the obesity group. Among patients in the obesity group, 36 (36.4%) were mildly obese (BMI 28.0–32.5 kg/m^2^), 38 (38.4%) were moderately obese (BMI 32.5–37.5 kg/m^2^), and 25 (25.3%) were severely obese (BMI 37.5–50 kg/m^2^). There were no significant differences between the two groups in terms of age, history of vaginal delivery, history of abdominal surgeries, uterine weight, or surgical indications. However, BMI was significantly higher in the obesity group compared to the control group. The baseline characteristics are summarized in Table 1.

**Table 1:** Comparison of baseline characteristics between the control and obese groups.

Variable	Control group (*n* = 112)	Obese group (*n* = 99)	*t*/*χ*^2^ value	*p*-value
Age (years)	49.30 ± 10.35	51.78 ± 11.03	*t* = 1.684	0.093
No vaginal delivery (*n*, %)	32 (28.57%)	31 (31.31%)	*χ*^2^ = 0.189	0.664
Previous abdominal surgery (*n*, %)	37 (33.04%)	30 (30.30%)	*χ*^2^ = 0.181	0.670
Uterine volume(cm^3^)	190.33 ± 122.67	209.61 ± 104.69	*t* = 1.219	0.224
Uterine weight (g)	201.07 ± 137.98	222.15 ± 129.68	*t* = 1.139	0.256
BMI (kg/m^2^)	21.87 ± 2.91	33.96 ± 3.99	*t* = 25.339	<0.001**
Surgical indication (n, %)				
- Uterine fibroids	69 (61.61%)	64 (64.65%)	*χ*^2^ = 1.544	0.672
- Endometrial atypical hyperplasia	28 (25.00%)	27 (27.27%)		
- Adenomyosis	2 (1.78%)	1 (1.01%)		
- Others	13 (11.61%)	7 (7.07%)		

Data are presented as mean ± standard deviation for continuous variables or *n* (%) for categorical variables. Statistical significance: *p* < 0.05 (*), *p* < 0.01 (**).

### 6.2 Intraoperative outcomes

The operative time was significantly longer in the obesity group compared to the control group. However, there were no significant differences between the two groups in terms of intraoperative blood loss, that is, the number of cases converted to other surgical approaches or intraoperative blood transfusion rates. These results are summarized in Table 2.

**Table 2:** Comparison of intraoperative parameters between the control and obese groups.

Variable	Control group (*n* = 112)	Obese group (*n* = 99)	*t*/*χ*^2^ value	*p*-value
Operative time (min)	101.75 ± 39.03	144.92 ± 63.81	*t* = 6.002	<0.001**
Intraoperative blood loss (ml)	137.90 ± 59.32	145.61 ± 49.37	*t* = 1.018	0.309
Conversion to other surgical route (*n*, %)	6 (5.35%)	5 (5.05%)	*χ*^2^ = 0.010	0.920
- To conventional laparoscopy	4 (3.57%)	4 (4.04%)	*χ*^2^ = 0.032	0.859
- To laparotomy	2 (1.78%)	1 (1.01%)	*χ*^2^ = 0.226	0.635
Reasons for conversion				
- Poor exposure	3 (2.68%)	3 (3.03%)	*χ*^2^ = 0.024	0.878
- Pelvic adhesions	3 (2.68%)	2 (2.02%)	*χ*^2^ = 0.098	0.754
Intraoperative blood transfusion (*n*, %)	4 (3.57%)	6 (6.06%)	*χ*^2^ = 0.721	0.396

Data are presented as mean ± standard deviation for continuous variables or n (%) for categorical variables. Statistical significance: *p* < 0.05 (*), *p* < 0.01 (**).

A multivariable linear regression analysis (Table 3) demonstrated that obesity was independently associated with a significant increase in operative time. However, no significant associations were observed between obesity and intraoperative blood loss.

**Table 3:** Multivariable linear regression results.

Outcome	*β* Coefficient	95% CI	*p*-value
Operative time (min)	39.2	24.5–53.9	<0.001**
Intraoperative blood loss (mL)	7.7	−5.4–20.8	0.245
Time to first flatus (h)	5.8	1.1–10.5	0.018*
Hemoglobin decrease (g/L)	0.53	−1.8–2.9	0.669
Length of hospital stay (d)	1.3	0.5–2.1	0.002**
Total cost (CNY¥10,000)	0.04	−0.01–0.09	0.073

Statistical significance: *p* < 0.05 (*), *p* < 0.01 (**).

For categorical outcomes, the logistic regression analysis (Table 4) revealed no significant differences in the odds of conversion to other surgical approaches or intraoperative blood transfusion.

**Table 4:** Logistic regression results.

Outcome	Adjusted OR	95% CI	*p*-value
Conversion to another surgical route	0.94	0.31–2.83	0.920
Intraoperative blood transfusion	1.72	0.45–6.55	0.425
Urinary retention	1.28	0.53–3.11	0.597
Postoperative fever	0.97	0.42–2.23	0.933

### 6.3 Perioperative outcomes

Perioperative outcomes showed significant differences in terms of time to first flatus and length of hospital stay between the two groups. However, no significant differences were observed in urinary retention, postoperative fever, hemoglobin drop, or total hospitalization costs.

Importantly, no intraoperative or early postoperative injuries to surrounding organs were identified in this cohort of 211 patients. Specifically, there were no cases of bladder injury, ureteral injury, bowel serosal tears, or vaginal/vulvar trauma. In addition, no vaginal cuff hematomas, cuff infections, or symptomatic urinary tract infections were documented within the first postoperative week. These results are presented in Table 5.

**Table 5:** Comparison of perioperative parameters between the control and obese groups.

Variable	Control group (*n* = 112)	Obese group (*n* = 99)	t/*χ*^2^ value	*P*-value
Time to first flatus (h)	40.00 ± 16.87	46.28 ± 20.07	*t* = 2.468	0.014*
Urinary retention (*n*, %)	10 (8.92%)	11 (11.11%)	*χ*^2^ = 0.279	0.597
Postoperative fever (*n*, %)	14 (12.50%)	12 (12.12%)	*χ*^2^ = 0.007	0.933
Hemoglobin decrease (g/L)	12.06 ± 9.55	12.59 ± 8.29	*t* = 0.427	0.669
Length of hospital stay (d)	6.92 ± 3.11	8.43 ± 2.38	*t* = 3.921	<0.001**
Total cost (10,000 yuan)	1.84 ± 0.13	1.88 ± 0.19	*t* = 1.801	0.073

Data are presented as mean ± standard deviation for continuous variables or *n* (%) for categorical variables. Statistical significance: *p* < 0.05 (*), *p* < 0.01 (**).

The multivariable linear regression analysis (Table 3) further confirmed that obesity was independently associated with delayed time to first flatus and prolonged hospital stays. However, no significant associations were observed between obesity and hemoglobin decrease or total hospitalization costs.

For categorical outcomes, the logistic regression analysis (Table 4) revealed no significant differences in the odds of urinary retention or postoperative fever.

### 6.4 Perioperative outcomes among the BMI groups

Perioperative outcomes demonstrated significant differences among the three BMI groups in terms of operative time, time to first flatus, and hemoglobin drop. In contrast, no significant differences were observed among the groups in terms of intraoperative blood loss, length of hospital stay, or total hospitalization costs. Additionally, the incidence of perioperative complications, such as urinary retention, postoperative fever, intraoperative transfusion, and conversion to other surgical approaches, did not differ significantly between the groups. These findings are summarized in Table 6.

**Table 6:** Comparison of perioperative outcomes among the BMI groups.

Variable	Mildly obese (*n* = 36)	Moderately obese (*n* = 38)	Severely obese (*n* = 25)	*H*/*F*/*χ*^2^ value	*p*-value
Operative time (min)	141.9 (105.6–192.3)	156.9 (116.0–223.9)	178.9 (135.2–251.8)	*H* = 10.223	0.006*
Intraoperative blood loss (mL)	150 (100–210)	160 (120–230)	180 (120–240)	*H* = 5.991	0.050
Time to first flatus (h)	41.2 (32.3–55.9)	49.6 (35.7–61.8)	55.9 (38.1–83.2)	*H* = 8.610	0.013*
Hemoglobin decrease (g/L)	7.09 (3.61–10.77)	9.48 (5.75–15.70)	13.21 (8.21–23.61)	*H* = 6.260	0.044*
Length of hospital stay (d)	8.74 ± 2.19	8.92 ± 2.11	8.69 ± 2.02	*F* = 0.172	0.843
Total cost (10,000 yuan)	1.82 (1.62–2.07)	1.88 (1.73–2.11)	1.91 (1.78–2.19)	*H* = 4.910	0.086
Conversion to other approach (*n*, %)	2 (5.6%)	3 (7.9%)	0 (0.0%)	*χ*^2^ = 2.865	0.238
Intraoperative transfusion (*n*, %)	2 (5.6%)	2 (5.3%)	2 (8.0%)	*χ*^2^ = 0.271	0.873
Urinary retention (*n*, %)	4 (11.1%)	5 (13.2%)	2 (8.0%)	*χ*^2^ = 0.531	0.765
Postoperative fever (*n*, %)	5 (13.9%)	4 (10.5%)	3 (12.0%)	*χ*^2^ = 0.103	0.950

Data are presented as mean ± standard deviation for continuous variables or *n* (%) for categorical variables. Statistical significance: *p* < 0.05 (*), *p* < 0.01 (**).

## 7 Discussion

### 7.1 Obesity and its impact on surgical outcomes

Obesity has emerged as a critical global health challenge, with its prevalence rising significantly in recent years (9, 10). According to Chinese standards, a body mass index (BMI) ≥ 28 kg/m^2^ is classified as obesity (11). Although the World Health Organization (WHO) recommends a lower cutoff of 27.5 kg/m^2^ for Asian populations, the 28 kg/m^2^ threshold is widely used in Chinese studies, as it better reflects obesity-related health risks in the Chinese population (11, 12).

Beyond its impact on the quality of life, obesity is closely associated with various chronic diseases, including cardiovascular diseases, diabetes, and certain cancers (9). Additionally, obesity can lead to hormonal imbalances and metabolic abnormalities, increasing the risk of reproductive system disorders such as endometrial polyps, uterine fibroids, endometrial hyperplasia, and endometrial cancer (1, 4, 5, 13). These conditions often necessitate total hysterectomy (TH), and obese women consequently represent a population with heightened clinical and surgical risks (4, 7).

### 7.2 The role of v-NOTES in obese patients

v-NOTES is an advanced minimally invasive surgical technique that offers advantages such as reduced trauma, faster postoperative recovery, lower pain levels, and the absence of abdominal scars (2, 3). However, its application in obese patients poses challenges due to anatomical constraints, such as a narrow vaginal canal and excessive pelvic and abdominal fat, raising concerns about its feasibility and efficacy in this population (5). While previous studies have demonstrated the benefits of v-NOTES in treating benign gynecological diseases, limited research has directly compared surgical outcomes between obese and non-obese patients undergoing v-NOTES for TH (14–16). Notably, although our study focuses on benign conditions, the complexity of obesity as a clinical factor has also been highlighted in malignant contexts, where the so-called ‘obesity paradox’—suggesting potential survival benefits in certain cancers—has been investigated but not confirmed (17).

### 7.3 Key findings and their implications

Our study retrospectively analyzed 211 patients who underwent v-NOTES TH, dividing them into obesity and control groups based on BMI. The results showed no significant differences between the two groups in terms of intraoperative blood loss, conversion to other surgical approaches, intraoperative blood transfusion, postoperative urinary retention, postoperative fever, hemoglobin drop, or total hospitalization costs. These findings indicate that v-NOTES is a safe and feasible surgical option for obese patients, aligning with prior studies (9, 18).

However, the study identified significant differences in operative time, time to first flatus, and length of hospital stay, which were all longer in the obesity group. The multivariable regression analysis confirmed that obesity was independently associated with these outcomes. Further subgroup analysis within the obesity group revealed that higher BMI was significantly associated with prolonged operative time, delayed gastrointestinal recovery, and greater hemoglobin drop (all *p* < 0.05). These findings suggest a dose–response relationship between BMI and surgical complexity, even among obese patients. As BMI increases, anatomical challenges and metabolic alterations may further impair surgical efficiency and recovery. These results underscore the need for stratified perioperative planning and enhanced intraoperative vigilance when managing patients with more severe obesity.

Although the incidence of postoperative urinary retention was not statistically different between the two BMI groups, the overall rate (~11%) appears relatively high. This complication is likely multifactorial in origin (19). In v-NOTES procedures, intraoperative traction or irritation of the bladder and pelvic nerves may temporarily impair voiding function. Additionally, some patients received opioid-based analgesics, which are known to inhibit detrusor muscle activity and delay micturition. Other potential contributing factors include postoperative pain, anxiety, delayed mobilization, and timing of catheter removal. These mechanisms may act synergistically to increase the risk of transient urinary retention after surgery (20).

### 7.4 Factors contributing to prolonged operative time

The prolonged operative time observed in obese patients can be attributed to multiple anatomical and technical challenges. Excessive abdominal and visceral fat may obscure the surgical field, hinder instrument manipulation, and complicate visualization of target structures. Additionally, a narrow vaginal canal in some obese patients may further restrict surgical access and increase the technical complexity of the procedure. These factors collectively contribute to the longer operative times noted in this study.

### 7.5 Delayed gastrointestinal recovery in obese patients

Delayed gastrointestinal recovery, as indicated by prolonged time to first flatus, was another notable finding among obese patients. Several mechanisms may account for this phenomenon. First, prolonged operative time in obese patients can lead to greater tissue trauma and heightened inflammatory responses, which increase the release of cytokines such as interleukin-8 and tumor necrosis factor-alpha—both known to impair gastrointestinal motility (21). Additionally, extended exposure to anesthesia may suppress the autonomic regulation of the digestive system, further delaying recovery of bowel function (22).

Obesity itself is associated with slower gastric emptying and reduced intestinal motility (23). Moreover, obese individuals often exhibit elevated levels of baseline systemic inflammation and metabolic dysfunction. These conditions may be exacerbated by surgical stress, resulting in hormonal imbalances that disrupt gastrointestinal motility and appetite regulation, particularly involving hormones such as insulin (24). Finally, psychological factors may also play a role. Obese patients may experience greater postoperative anxiety or mood disturbances, which could reduce adherence to early mobilization, dietary recommendations, and overall recovery plans—further contributing to delayed gastrointestinal function (25).

### 7.6 Strengths and limitations

This study has several strengths. First, the use of multivariable regression analysis allowed us to adjust for potential confounders such as age, uterine weight, and surgical indications, thereby enhancing the validity of our findings. Second, the study provides valuable insights into the feasibility and safety of v-NOTES in obese patients, a population often underrepresented in surgical research.

However, several limitations must be acknowledged. As a retrospective study, it is subject to inherent biases, such as unmeasured confounders, including surgeon experience and variations in surgical technique. Additionally, the relatively small sample size may reduce the generalizability of the findings. Moreover, long-term outcomes, such as recurrence rates or quality of life, were not assessed.

However, several limitations must be acknowledged. As a retrospective study, it is subject to selection bias and unmeasured confounders, including surgeon experience and variations in surgical technique. The relatively small sample size may limit generalizability, and long-term outcomes were not assessed.

Moreover, postoperative analgesia protocols were not standardized across the three participating centers: not all patients received analgesic pumps, and the drug regimens varied. As such, we were unable to systematically evaluate the impact of analgesic strategies on outcomes such as urinary retention. Future multicenter prospective studies with standardized perioperative management are needed to validate these findings.

## 8 Conclusion

v-NOTES is a safe and feasible surgical approach for obese patients undergoing TH, with a comparable safety profile to non-obese patients. However, obesity is independently associated with prolonged operative time, delayed gastrointestinal recovery, and extended hospital stays. These findings underscore the importance of tailoring perioperative and postoperative care strategies to address the unique needs of obese patients. Interventions to promote gastrointestinal recovery and reduce hospital stays may be particularly beneficial. Future research should focus on validating these findings in larger, multicenter cohorts and exploring novel strategies to optimize outcomes for obese patients undergoing minimally invasive surgery.

## Data Availability

The raw data supporting the conclusions of this article will be made available by the authors, without undue reservation.
